# Biological Activity of Pumpkin Byproducts: Antimicrobial and Antioxidant Properties

**DOI:** 10.3390/molecules27238366

**Published:** 2022-11-30

**Authors:** Maria G. Leichtweis, Adriana K. Molina, Tânia C. S. Pires, Maria Inês Dias, Ricardo Calhelha, Khaldoun Bachari, Borhane E. C. Ziani, M. Beatriz P. P. Oliveira, Carla Pereira, Lillian Barros

**Affiliations:** 1 Centro de Investigação de Montanha (CIMO), Instituto Politécnico de Bragança, Campus de Santa Apolónia, 5300-253 Bragança, Portugal; 2 Laboratório Associado para a Sustentabilidade e Tecnologia em Regiões de Montanha (SusTEC), Instituto Politécnico de Bragança, 5300-253 Bragança, Portugal; 3 Centre de Recherche Scientifique et Technique en Analyses Physico-Chimiques-CRAPC, Bou Ismaïl 42004, Algeria; 4 REQUIMTE—Science Chemical Department, Faculty of Pharmacy, University of Porto, Rua Jorge Viterbo Ferreira no. 228, 4050-313 Porto, Portugal

**Keywords:** biologically active compounds, phenolic profile, antimicrobial activity, antioxidant activity, pumpkin byproducts, bio-based food preservatives

## Abstract

Pumpkin fruits are widely appreciated and consumed worldwide. In addition to their balanced nutritional profile, pumpkin species also present valuable bioactive compounds that confer biological and pharmacological properties to them. However, the seeds, peels, and fibrous strands resulting from pumpkin processing are still poorly explored by the food industry. The current study used those fruit components from the genotypes of pumpkin that are economically significant in Portugal and Algeria to produce bioactive extracts. In order to support their usage as preservatives, their phenolic content (HPLC-DAD-ESI/MS) and antioxidant (OxHLIA and TBARS) and antimicrobial properties (against eight bacterial and two fungal strains) were assessed. In terms of phenolic profile, the peel of the Portuguese ‘Common Pumpkin’ showed the most diversified profile and also the highest concentration of total phenolic compounds, with considerable concentrations of (-)-epicatechin. Regarding the antioxidant capacity, the seeds of ‘Butternut Squash’ from both countries stood out, while the fibrous strands of Portuguese ‘Butternut Squash’ and the seeds of Algerian ‘Gold Nugget Pumpkin’ revealed the strongest antimicrobial activity. The bioactive compounds identified in the pumpkin byproducts may validate their enormous potential as a source of bio-based preservatives that may enhance consumers’ health and promote a circular economy.

## 1. Introduction

Natural matrices have been increasingly investigated as sources of bioactive molecules, not only for their benefits for human health, but also for their technological functionalities in food and cosmetic products [1,2,3,4,5]. Their wealth in such compounds has been widely demonstrated along the last decades, as have their bioactive properties. Nevertheless, with the current lifestyle of modern societies, vegetable-based meals are often limited to practical solutions as ready-to-use foodstuffs. With the increasing demand for these products, a considerable amount of byproducts is generated in the food industry, where distinct parts of plants, vegetables, and fruits are simply discarded along the process.

To promote the sustainability of these processes, recent studies have been focusing on the recovery of distinct byproducts for the extraction of high value-added compounds [6,7]. As examples, a great profile of bioactive compounds was reported for the extracts of sweet potato leaves, which were mainly composed of phenolic compounds with related antioxidant, anti-mutagenic, antidiabetic, anticancer, and anti-inflammatory activity, as well as heart and hepatoprotection properties [6]. In another study, antibacterial and antioxidant activities were shown by the betacyanin-rich extracts of red pitaya peels [7].

A common process in the food industry is the production of pumpkin pulp formulations, which generates high volumes of byproducts such as peels, seeds, and fibrous strands. This fruit is appreciated worldwide for its pleasant taste and nutritional properties, and is a source of carbohydrates, protein, fat, vitamins, and minerals. Additionally, it also presents diuretic, antirheumatic, stimulant, anti-inflammatory, antidiabetic, antidepressant, and antioxidant properties, among many other beneficial effects well-reported in the literature [8,9,10,11,12]. Despite the fact that distinct parts of the fruit can be consumed, the pulp is more appreciated, while the byproducts are often discarded or underutilized. However, these fruit parts can present important contents of value-added compounds such as minerals, polyunsaturated fatty acids, tocopherols, polyphenols, carotenoids, and phytosterols [11,13,14,15,16]. For instance, *Cucurbita pepo* species seed oil from Pakistan revealed high nutritional components, including proteins, minerals, and unsaturated fatty acids, mainly linoleic and oleic acids, in addition to effective inhibitory activity against the gram positive bacterium *Staphylococcus aureus* [17]. In another study, *Curcubita maxima* seed oil compounds were associated with high protection against oxidative stress, among which six phenolic compounds were detected, with the prevalence of syringic acid, in addition to tocopherols and sterols, especially δ-tocopherol and β-sisosterol, respectively [18]. Pumpkin skin and seeds were also reported as presenting high contents of total phenolic compounds and a strong antioxidant potential, evaluated through different chemical assays [19]. Moreover, pumpkin rinds and seeds were applied to bakery products to increase their antioxidant capacity and total phenolic concentration [20]. Given the chemical composition of these byproducts and their important antioxidant and antimicrobial capacities, they could find useful application in the development of natural food preservatives.

In order to scientifically demonstrate this potential, the current study evaluated the byproducts (peels, fibrous strands, and seeds) from three Portuguese (‘Butternut Squash’, ‘Common Pumpkin’, and ‘Kabocha Squash’) and three Algerian (‘Butternut Squash’, ‘Gold Nugget Pumpkin’, and ‘Musquée de Provence’) genotypes of pumpkins. The HPLC-DAD/ESI-MS was used to investigate the hydroethanolic extracts’ phenolic content, while further bioactivities, namely the antioxidant, antibacterial, antifungal, and cytotoxic properties, were assessed in order to determine their potential to be used as natural food preservatives.

## 2. Results and Discussion

### 2.1. Phenolic Compounds Profile

**Figure 1:**
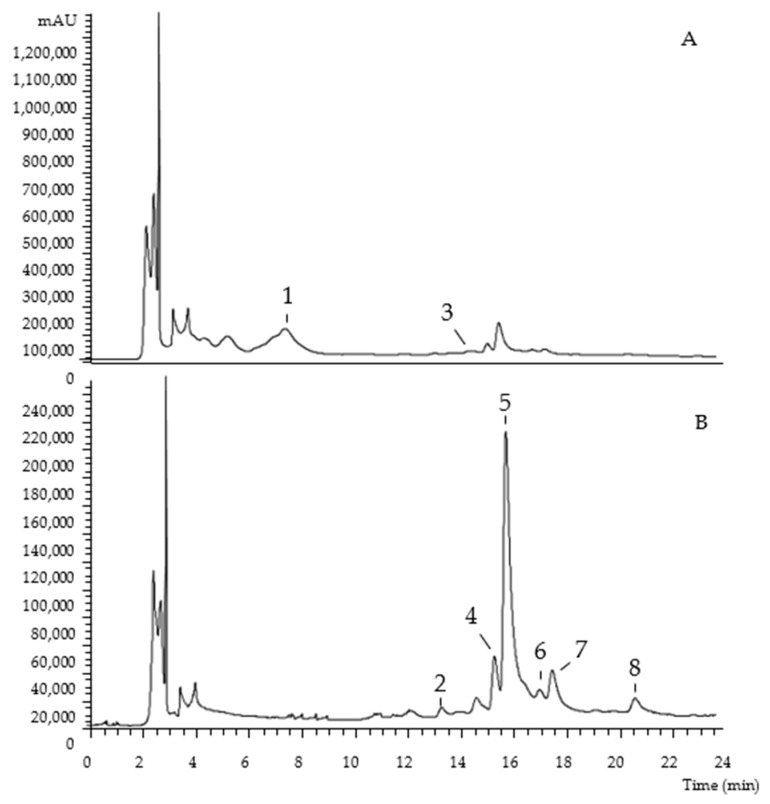
Portuguese ‘Common Pumpkin’ seeds chromatogram, recorded at 280 nm (**A**) and 370 nm (**B**).

**Figure 2:**
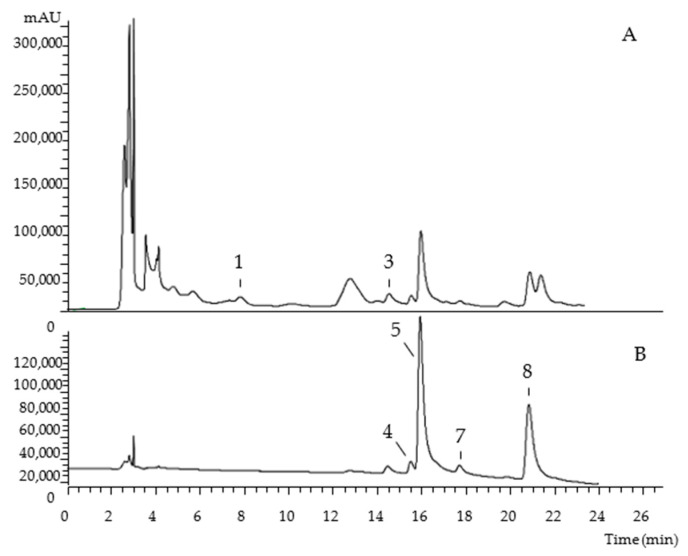
Algerian ‘Gold Nugget Pumpkin’ peel chromatogram, recorded at 280 nm (**A**) and 370 nm (**B**).

The chromatographic characterization of phenolic compounds, regarding UV-vis at the maximum absorption, deprotonated ion, mass fragmentation, and tentative identification of the hydroethanolic extracts of Portuguese and Algerian pumpkin byproducts are described in Table 1. Eight compounds were found, belonging to the phenolic acids (peak 3), flavan-3-ols (peak 1), and flavonoids (peaks 2, 4, 5, 6, 7, and 8) families. As examples, Figure 1 and Figure 2 show the phenolic profile obtained for Portuguese ‘Common Pumpkin’ seeds and Algerian ‘Gold Nugget Pumpkin’ peel, respectively.

Peak 3 presented a deprotonated ion [M-H]^−^ at *m/z* 405 and a major MS^2^ fragment at *m/z* 281 that corresponded to the loss of the 4-hydroxybenzyl alcohol moiety (124 Da); it also produced MS^2^ fragments at *m/z* 137 (hydroxybenzoic acid) and *m/z* 93 (loss of glucosyl residue and CO_2_). These chromatographic responses were in accordance with those previously described by Jaiswal & Kuknert [21] and the peak was tentatively identified as 7 4-*O*-(6′-*O*-glucosyl-4″-hydroxybenzoyl)-4-hydroxybenzyl alcohol. It is also important to state that this compound was found in *Lagenaria siceraria* Stand. (Bottle Gourd) [21] that belong to the *Cucurbitaceae* family, as pumpkins.

Peak **1** ([M-H]^−^ at *m/z* 289) was identified as (-)-epicatechin by comparing the retention time, UV-vis at the maximum absorption (λ_máx_ 280 nm), and mass spectra with the available standard compound. These compounds were previously described in *Cucurbita moschata* samples from Australia [19].

The family of flavonoids was the most abundant in terms of the number of compounds detected, mainly *O*-glycosylated derivatives of quercetin, kaempferol, and isorhamnetin, as previously described by Iswaldi et al. [22] in *Cucurbita pepo* L. The detected compounds could be divided in two groups, the first one presented two sugar moieties linked to the flavonoid aglycone (peaks 6, 7, and 8), and the second one presented three sugar moieties (peaks 2, 4, and 5). Peaks 6 ([M-H]^−^ at *m/z* 593) and 7/8 ([M-H]^−^ at *m/z* 623) presented only one MS^2^ fragment at *m/z* 285 (kaempherol aglycone) and *m/z* 315 (isorhamnetin aglycone), respectively, corresponding to the jointed loss of a deoxyhexosyl and hexosyl moiety ([M-H-146-162]^−^), being tentatively identified as kaempferol-*O*-deoxyhexosyl-hexoside and isorhamnetin-*O*-deoxyhexosyl-hexoside, respectively. Finally, peaks 2 ([M-H]^−^ at *m/z* 775), 4 ([M-H]^−^ at *m/z* 739), and 5 ([M-H]^−^ at *m/z* 769) also presented a unique MS^2^ fragment at *m/z* 301 (quercetin aglycone), *m/z* 285, and *m/z* 315, respectively, that corresponded to the loss of two deoxyhexosyl moieties and one hexosyl moiety ([M-H-146-146-162]^−^), being tentatively identified as quercetin-*O*-dideoxyhexosyl-hexoside, kaempferol-*O*-dideoxyhexosyl-hexoside, and isorhamnetin-*O*-dideoxyhexosyl-hexoside, respectively.

As shown in Table 2, concerning the Portuguese samples, the ‘Common Pumpkin’ peel presented the statistically higher (*p* < 0.05) total of phenolic compounds (9.4 ± 0.3 mg/g of extract), followed by the fiber of ‘Kabocha Squash’ (4.8 ± 0.1 mg/g of extract) and the peel of ‘Butternut Squash’ (4.73 ± 0.01 mg/g of extract), the values of which did not differ significantly (*p* > 0.05). These totals are mainly comprised of the flavan-3-ols and flavonoids families, while phenolic acids are not representative or were not detected. The (-)-epicatechin (Peak 1) was the most abundant compound of all the samples evaluated. Epicatechin was also reported to be the major constituent in *Momordica caranthia* (bitter melon) [23], which belongs to the same family as pumpkins.

Different profiles were seen in the samples from Algeria (Table 3), where more expressive contents of phenolic acids were found in the ‘Gold Nugget Pumpkin’ fibrous strands (2.27 ± 0.02 mg/g of extract), while flavonoids are the most representative compounds of the total phenolic compounds were found in all the peels and in the seeds of ‘Musquée de Provence’. High levels of phenolic acids and flavonoids were also reported by Mokhtar et al. [24], in mature pumpkins (*Cucurbita moschata* Duchesne). The ‘Gold Nugget Pumpkin’ peel presented the highest value of total phenolic compounds, followed by the fibrous strands of this genotype (4.1 ± 0.1 and 3.93 ± 0.05 mg/g of extract, respectively), being statistically different (*p* < 0.05) from each other. Furthermore, peak 2 and 6 were not found in the Algerian extracts and no peak was identified in the extracts of ‘Gold Nugget Pumpkin’ and ‘Butternut Squash’ seeds. To the best of our knowledge, this is the first report on the phenolic composition of these pumpkin genotype byproducts generated in the food industry.

### 2.2. Antioxidant Acitivity

The bioactive capacity of the pumpkin byproducts was assessed in order to evaluate the preservative potential of their hydroethanolic extracts. The antioxidant capacity was analyzed through two cell-based assays, which present the advantage of evaluating oxidizable biological targets. The samples presented great antioxidant results, shown in Table 4 and Table 5, in the two mechanisms evaluated: the inhibition of oxidative hemolysis (OxHLIA) in sheep erythrocytes suspension and the inhibition of lipid peroxidation (TBARS) in porcine brain homogenates. 

Regarding the Portuguese pumpkin genotype extracts (Table 1), the seeds presented the best results in the TBARS assay, especially the ‘Kabocha Squash’ (IC_50_: 164 ± 8 μg/mL) and the ‘Butternut Squash’ (IC_50_: 185 ± 7 μg/mL) genotypes. These results did not differ significantly (*p* > 0.05) from the positive control Trolox, which represents great results for a natural extract. In the OxHLIA assay, the results were quite similar among the samples. The IC_50_ values ranged from 43 to 96 μg/mL, which represent about 2 to 4.5-fold higher concentrations than that of Trolox (21.8 μg/mL), except for the ‘Common Pumpkin’ fibrous strands and the ‘Kabocha Squash’ peel, which presented higher (*p* < 0.05) IC_50_ values (365 ± 13 μg/mL and 209 ± 10 μg/mL, respectively). In fact, samples from Portugal showed greater anti-hemolytic capacity than those from Algeria (Table 2), which presented IC_50_ values from 115 ± 6 μg/mL to 588 ± 18 μg/mL. Interestingly, despite not presenting anti-hemolytic properties, the seeds of ‘Gold Nugget Pumpkin’ revealed the strongest lipid peroxidation inhibition capacity (91 ± 4 μg/mL), which can possibly be explained by the interference of other compounds, such as lipids, that have a great influence in the OxHLIA assay [25].

These results are in agreement with the literature, where some authors also reported the antioxidant capacity of different pumpkin parts, mostly the seeds, through different methods. Akomolafe et al. [26] evaluated the antioxidant potential of pumpkin (*Cucurbita pepo* L.) seeds and reported that their methanolic extract caused a notable reduction in the TBARS produced in albino rat’s testicular tissue. In another study [27], pumpkin seeds and shells extracted with different solvents presented a great capacity for DPPH radical scavenging. The most efficient solvent was the 70% ethanol and the shell samples presented the higher inhibition percentage, reaching up to 71.0 ± 0.97% of DPPH radicals inhibition. Furthermore, in a study assessing the incorporation of pumpkin seeds into chicken burgers, the lipid stability during storage and the antioxidant properties were improved when compared to the raw burgers [28]. The results found in the literature were not comparable to the ones presented herein given the difference in the parts of the plant used and the different methods employed. In the present study, only cell-based methods were applied in order to better mimic the mechanisms involved in in vivo systems.

### 2.3. Antimicrobial and Antifungal Activity

The microorganisms used in this assay are important food contaminants that can affect the quality of foodstuffs by deterioration and organoleptic damage and/or also affect the consumers’ health, causing intoxication and infection with serious related complications. The extracts obtained from pumpkin byproducts were capable of inhibiting the growth of at least two of the eight bacterial strains and one of the two fungal strains assessed.

Table 6, Table 7, Table 8 and Table 9 present the antimicrobial (antibacterial and antifungal) capacity of the Portuguese and Algerian samples. All of the samples from Portugal exhibited inhibition capacity against *Yersinia enterocolitica*; while the ones from Algeria inhibited *Staphylococcus aureus*. In terms of food preservation, these are important results because, according to the EFSA Journal, yersiniosis is recognized as the third most common zoonotic disease in the EU and, on the other hand, coagulase-positive *Staphylococcus* spp was found in a considerable number of food samples reported by Bulgaria, Italy, and Spain [29]. More specifically, *Y. enterocolitica* was found in 2.33% of retail food samples from China [30], being responsible for diarrhea, abdominal pain, and fever in consumers. In turn, *S. aureus* is the main representative of coagulase-positive *Staphylococcus* spp in food and, recently, its presence was reported in cold meals, mostly in salads served in the university canteens of northern Portugal [31]. Regarding fungi, the pumpkin byproduct extracts were tested against *Aspergillus*, which is an important fungus genus in food for causing its deterioration and producing mycotoxins. In fact, *Aspergillus brasiliensis* is a target microorganism in the validation of food packaging sterilization and *Aspergillus fumigatus* is considered as the most important filamentous fungal human pathogens [32,33]. All samples revealed the capacity to inhibit *A. brasiliensis* growth and the fibrous strands of Algerian pumpkins also protected against *A. fumigatus*, as well as the ‘Gold Nugget Pumpkin’ peel.

Furthermore, both samples of ‘Butternut Squash’ fibrous strands from Portugal and Algeria presented activity against the eight tested bacterial strains and six of the eighteen samples inhibited seven bacterial strains growth. The samples presented inhibition capacity in concentrations ranging from 2.5 to 10 mg/mL, and none of the samples revealed bactericidal nor fungicidal capacity.

These results do not agree with those obtained by Saavedra et al. [27], where pumpkin shells and seeds extracted with different solvents (70% ethanol, 70% methanol, 70% acetone, water, and dichloromethane) did not present antibacterial capacity against *Pseudomonas aeruginosa*, *Escherichia coli*, *S. aureus*, and *Listeria monocytogenes* up to the maximum tested concentration of 10 mg/mL. These differences could be explained by the different extraction solvents employed, and in the case of shells, also for being a different part of the plants. Regarding the seeds, in the present study, the hydroethanolic extract of those from Portuguese ‘Kabocha Squash’ were able to inhibit *P. aeruginosa*, *E. coli*, and *S. aureus* in a concentration of 10 mg/mL. *S. aureus* was also inhibited by the same concentration of Algerian ‘Musquée de Provence’ seed extracts. Moreover, the seeds of ‘Gold Nugget Pumpkin’ from Algeria inhibited the growth of *E. coli* (MIC = 10 mg/mL), *L. monocytogenes* (MIC = 2.5 mg/mL), and *S. aureus* (MIC = 2.5 mg/mL). *S. aureus* was also inhibited by the Potuguese ‘Butternut Squash’ seeds (MIC = 10 mg/mL).

Similar results were also found in a study performed with pumpkin leaves [34], where their hydroethanolic extracts did not inhibit *E. coli*, *Shigella flexneri*, *Salmonella enterica*, *L. monocytogenes*, *S. aureus*, and *Bacillus subtilis* growth, until 10 mg/mL was reached. Despite the fact that the authors tested the same extract concentration, these results are not directly comparable to the ones obtained in the present study given the differences in the parts of the plant used. More recently [14], the oil obtained from indigenous pumpkin seeds (*Cucurbita maxima* Linn.) presented antibacterial activity against eight strains of *E. coli* and *Shigella sonnei*, with inhibition zones ranging from 10.66 ± 0.57 to 18 ± 1.0 mm, by using the disc diffusion method. Moreover, polysaccharides extracted from pumpkin pulp presented antimicrobial activity against three bacteria and three fungi. The highest inhibition zone was found against *E. coli*, followed by *S. aureus*, also inhibiting *P. aeruginosa*, *Aspergillus flavus*, *A. fumigatus*, and *Aspergillus niger*. It was not possible to compare these results with the ones obtained herein because different methods were employed, as were different microorganisms, but it is interesting to observe that seed-related products also presented antimicrobial properties similar to the seeds assessed in this study.

### 2.4. Cytotoxic Potential

The potential safety of the extracts obtained from the pumpkin byproducts was verified by assessing their toxicity in a primary culture of non-tumor porcine liver cells (PLP2). None of the samples revealed cytotoxic properties up to the maximum concentration of 400 μg/mL tested, which is commonly used for the assessment of possible toxic effects of natural extracts in non-tumor cells. This is an important first validation as, so far, no studies have been found regarding the toxic effects of pumpkin extracts. On the contrary, many studies reveled their cytoprotective and anticarcinogenic effects [35]. In addition to the non-hepatotoxic effect of the pumpkin extracts reported in this study, Abou Seif [36] demonstrated the capacity of pumpkin oil to protect the liver against alcohol-induced hepatotoxicity and oxidative stress in albino rats.

## 3. Materials and Methods

### 3.1. Sample Preparation

The ‘Butternut Squash’, ‘Common Pumpkin’, and ‘Kabocha Squash’ genotypes of pumpkin fruits cultivated in Portugal and ‘Butternut Squash’, ‘Gold Nugget Pumpkin’, and ‘Musquée de Provence’ cultivated in Algeria were obtained in local markets of both countries at the end of the summer season. The samples were prepared by separating the pulp from the by-products, which were divided into peels (thickness < 150 mm), fibrous strands, and seeds. The samples were then lyophilized (FreeZone 4.5, Labconco), crushed, and extracted by maceration. Briefly, 2 g of powdered sample was extracted with 60 mL of an ethanol solution (ethanol:water, 80:20) at room temperature, with magnetic stirring, for 60 min. The extracts were filtered and this procedure was repeated with the residue. For the combined extracts the ethanol was vacuum-evaporated at 45 °C, and the residual water was lyophilized to dryness for subsequent analyses.

### 3.2. Characterization of the Phenolic Compounds Profile

The phenolic composition was assessed by high performance liquid chromatography coupled to a diode array detector and electrospray ionization—mass spectrometry (HPLC-DAD-ESI/MS), following the methodology described by Barros et al. [37]. The chromatographic data were acquired using a Dionex Ultimate 3000 UPLC (Thermo Scientific, San Jose, CA, USA), coupled to a diode array detector (280 and 370 nm) and an electrospray ionization mass detector (Linear Ion Trap LTQ XL, Thermo Finnigan, San Jose, CA, USA), working in the negative mode. The chromatographic separation was performed using aWaters Spherisorb S3 ODS-2 C18 (3 m, 4.6 mm 150 mm, Waters, Milford, MA, USA) column at 35 °C. The identification was performed by comparison with available standards or literature data; the quantification was achieved using the equations presented in the table footnotes. The results are presented in mg/mL.

### 3.3. Bioactive Performance

To evaluate the antioxidant potential, two cell-based assays were applied, namely OxHLIA in sheep erythrocytes [38] and TBARS in porcine brain homogenates [39]. The extracts’ capacity to inhibit the oxidative hemolysis was assessed in sheep blood erythrocytes and the extract concentration able to promote a delay of 60 min on the hemolysis was calculated based on the Ht_50_ values of the hemolytic curves for each concentration of extract. The results were expressed as IC_50_ values (μg/mL), which give the extract concentration required to keep 50% of the erythrocyte population intact for 60 min. On the other hand, the capacity to inhibit the TBARS formation was tested using porcine brain cells as oxidizable biological substrates and the results were expressed as EC_50_ values (μg/mL), meaning the extract concentration responsible for 50% of antioxidant activity. Trolox was used as a positive control in both assays.The antimicrobial activity was tested against two fungi and eight bacteria of interest in food contamination, following the methodology described by Heleno et al. [40], using the p-iodonitrotetrazolium chloride (INT) method [41]. The microrganisms (Frilabo, Porto, Portugal) assessed were *Enterobacter cloacae* (ATCC 49741), *Escherichia coli* (ATCC 25922), *Pseudomonas aeruginosa* (ATCC 9027), *Salmonella enterica* subsp (ATCC 13076), *Yersinia enterocolitica* (ATCC 8610), *Bacillus cereus* (ATCC 11778), *Listeria monocytogenes* (ATCC 19111), *Staphylococcus aureus* (ATCC 25923), *Aspergillus fumigatus* (ATCC 204305), and *Aspergillus brasiliensis* (ATCC 16404). The results are presented as IC_50_ values, in mg/mL.

Furthermore, the cytotoxicity was tested in a primary culture of non-tumor porcine liver cells (PLP2) obtained from a freshly harvested porcine liver (purchased from a local slaughter house), by the Sulforhodamine B (SRB) colorimetric assay [42]. Ellipticine was used as positive control and the results were presented as IC_50_ values (extract concentration inhibiting 50% of the net cell growth), in μg/mL.

### 3.4. Statistical Analysis

All samples were analyzed in triplicate and the results were expressed as mean ± standard deviation. For the comparison of only two groups of data, a student’s *t*-test was applied, while for more groups, the one-way analysis of variance (ANOVA) was used. For that purpose, the normal distribution and the homogeneity of variance of data were evaluated by the Shapiro Wilk’s and Levene’s tests, respectively. The Tukey’s honestly significant difference (HSD) test was applied for homoscedastic data (*p* > 0.05) and Tamhane’s T2 multiple comparison test was for the heteroscedastic data. The tests were performed at a 5% significance level using SPSS Statistics software (IBM SPSS Statistics for Windows, Version 22.0. Armonk, NY, USA: IBM Corp).

## 4. Conclusions

The byproducts of different pumpkin genotypes were evaluated in terms of biologically active compounds. The phenolic profile was analyzed, with a tentative identification followed by quantification. It was also possible to verify that, despite the influence of the different genotypes of pumpkin, environmental and agronomic conditions between countries, and major and secondary metabolites composition, all samples presented bioactive properties, with the genotype ‘Butternut squash’ from both countries presenting the strongest antioxidant properties. In turn, Portuguese ‘Butternut squash’ fibrous strands and Algerian ‘Gold Nugget Pumpkin’ seeds presented a highest antimicrobial activity than the remaining byproducts. Regarding the phenolic profile, Portuguese ‘Common Pumpkin’ peel was the sample presenting the most diversified profile and the highest total content of phenolic compounds, among which (-)-epicatechin stood out. Along with the assessed phenolic compounds, many other compounds could be responsible for the bioactivities reported in this study; as also other bioactive properties could possibly be presented by the samples; however, this is an important first screening that corroborates the importance of reusing and recycling this kind of byproduct to be reintroduced in other steps of the production chain or even in other fields, such as pharmaceutics or cosmetics, for instance.

## Figures and Tables

**Table 1:** Phenolic compounds characterized by HPLC-DAD-ESI/MS in the different samples of pumpkin.

Peak	Rt (min)	λmax (nm)	[M-H]^−^ (*m/z*)	MS^2^ (*m/z*)	Tentative Identification
1	7.71	280	289	245 (100), 205 (45)	(-)-Epicatechin
2	13.42	345	775	301 (100)	Quercetin-*O*-dideoxyhexosyl-hexoside
3	14.73	263	405	281 (100), 137 (12), 93 (5)	7 4-*O*-(6′-*O*-Glucosyl-4″-hydroxybenzoyl)-4-hydroxybenzyl alcohol
4	15.42	344	739	285 (100)	Kaempferol-*O*-dideoxyhexosyl-hexoside
5	15.85	354	769	315 (100)	Isorhamnetin-*O*-dideoxyhexosyl-hexoside
6	17.12	348	593	285 (100)	Kaempferol-*O*-deoxyhexosyl-hexoside
7	17.6	365	623	315 (100)	Isorhamnetin-*O*-deoxyhexosyl-hexoside
8	20.73	365	623	315 (100)	Isorhamnetin-*O*-deoxyhexosyl-hexoside

**Table 2:** Quantification of the phenolic compounds found in the pumpkin samples from Portugal (mg/g of extract).

Peak	Common Pumpkin			Butternut Squash			Kabocha Squash		
	Peel	Fibrous Strands	Seeds	Peel	Fibrous Strands	Seeds	Peel	Fibrous Strands	Seeds
1	4.58 ± 0.08 ^a^	3.04 ± 0.05 ^b^	1.74 ± 0.03 ^f^	2.56 ± 0.03 ^d,e^	2.47 ± 0.07 ^e^	2.63 ± 0.02 ^c,d^	1.50 ± 0.07 ^g^	2.7 ± 0.1 ^c^	1.29 ± 0.05 ^h^
2	0.50 ± 0.02 ^b^	0.484 ± 0.006 ^b^	0.49 ± 0.02 ^b^	n.d.	n.d.	n.d.	n.d.	0.533 ± 0.003 ^a^	0.474 ± 0.001 ^b^
3	0.214 ± 0.009 ^a^	n.d.	n.d.	n.d.	n.d.	n.d.	0.116 ± 0.004 ^b^	n.d.	n.d.
4	0.65 ± 0.03 ^a^	0.487 ± 0.007 ^e^	0.458 ± 0.008 ^f^	0.6096 ± 0.0002 ^b^	n.d.	0.457 ± 0.008 ^f^	0.58 ± 0.02 ^c^	0.5218 ± 0.0004 ^d^	0.461 ± 0.003 ^e,f^
5	1.60 ± 0.08 ^a^	n.d.	n.d.	0.543 ± 0.004 ^c^	n.d.	0.454 ± 0.007 ^d^	0.62 ± 0.03 ^b^	0.494 ± 0.002 ^c,d^	n.d.
6	0.59 ± 0.03 ^a^	n.d.	n.d.	0.519 ± 0.005 ^b^	n.d.	n.d.	0.58 ± 0.03 ^a^	0.493 ± 0.003 ^b^	n.d.
7	0.69 ± 0.03 ^a^	n.d.	n.d.	0.496 ± 0.005 ^c^	n.d.	n.d.	0.56 ± 0.02 ^b^	n.d.	n.d.
8	0.55 ± 0.03 ^a^	n.d.	n.d.	n.d.	n.d.	n.d.	0.54 ± 0.03 ^a^	n.d.	n.d.
Total flavan-3-ols	4.58 ± 0.08 ^a^	3.04 ± 0.05 ^b^	1.74 ± 0.03 ^f^	2.56 ± 0.03 ^d,e^	2.47 ± 0.07 ^e^	2.63 ± 0.02 ^c,d^	1.50 ± 0.07 ^g^	2.7 ± 0.1 ^c^	1.29 ± 0.05 ^h^
Total phenolic acids	0.214 ± 0.009 ^a^	n.d.	n.d.	n.d.	n.d.	n.d.	0.116 ± 0.004 ^b^	n.d.	n.d.
Total flavonoids	4.6 ± 0.2 ^a^	0.97 ± 0.01 ^d^	0.95 ± 0.03 ^d^	2.17 ± 0.01 ^c^	n.d.	0.91 ± 0.02 ^d^	2.9 ± 0.1 ^b^	2.042 ± 0.003 ^c^	0.934 ± 0.002 ^d^
Total phenolic compounds	9.4 ± 0.3 ^a^	4.01 ± 0.06 ^d^	2.693 ± 0.004 ^f^	4.73 ± 0.01 ^b,c^	2.47 ± 0.07 ^f^	3.538 ± 0.007 ^e^	4.50 ± 0.06 ^c^	4.8 ± 0.1 ^b^	2.23 ± 0.04 ^g^

n.d.—not detected. Calibration curves used for quantification: (-)-catequin (*y* = 84,950*x* − 23,200, *R*^2^ = 0.999, LOD = 0.17 μg/mL; LOQ = 0.68 μg/mL, peak 1); *p*-hydroxybenzoic acid (*y* = 208,604*x* + 173056, *R*^2^ = 0.9995, LOD = 1.37 μg/mL; LOQ = 4.15 μg/mL, peak 3); and quercetin 3-*O*-glucoside (*y* = 34,843*x* − 160,173; *R*^2^ = 0.9998; LOD = 0.21 μg/mL; LOQ = 0.71 μg/mL, peaks 2, 4, 5, 6, 7 and 8). ANOVA analysis—In each row different letters mean significant differences (*p* < 0.05).

**Table 3:** Quantification of the phenolic compounds found in the pumpkin samples from Algeria (mg/g of extract).

Peak	Gold Nugget Pumpkin			Butternut Squash			Musquée de Provence		
	Fibrous Strands	Seeds	Peel	Fibrous Strands	Seeds	Peel	Fibrous Strands	Seeds	Peel
1	1.66 ± 0.07 ^a^	n.d.	0.51 ± 0.02 ^e^	0.97 ± 0.03 ^c^	n.d.	0.346 ± 0.009 ^f^	1.13 ± 0.01 ^b^	n.d.	0.577 ± 0.008 ^d^
3	2.27 ± 0.02 ^a^	n.d.	0.134 ± 0.006 ^d^	0.176 ± 0.006 ^c^	n.d.	tr.	0.207 ± 0.007 ^b^	0.0762 ± 0.0002 ^e^	n.d.
4	n.d.	n.d.	0.94 ± 0.05 ^a^	0.462 ± 0.002 ^c^	n.d.	0.4526 ± 0.0003 ^c^	0.4790 ± 0.0007 ^b^	n.d.	0.476 ± 0.003 ^b^
5	n.d.	n.d.	1.65 ± 0.07 ^a^	n.d.	n.d.	0.44904 ± 0.00006 ^b^	n.d.	0.460 ± 0.005 ^b^	0.474 ± 0.002 ^b^
7	n.d.	n.d.	n.d.	n.d.	n.d.	0.4418 ± 0.0001	n.d.	n.d.	n.d.
8	n.d.	n.d.	0.85 ± 0.02	n.d.	n.d.	n.d.	n.d.	n.d.	n.d.
Total flavan-3-ols	1.66 ± 0.07 ^a^	n.d.	0.51 ± 0.02 ^e^	0.97 ± 0.03 ^c^	n.d.	0.346 ± 0.009 ^f^	1.13 ± 0.01 ^b^	n.d.	0.577 ± 0.008 ^d^
Total phenolic acids	2.27 ± 0.02 ^a^	n.d.	0.134 ± 0.006 ^d^	0.176 ± 0.006 ^c^	n.d.	tr.	0.207 ± 0.007 ^b^	0.0762 ± 0.0002 ^e^	n.d.
Total flavonoids	n.d.	n.d.	3.4 ± 0.1 ^a^	0.462 ± 0.002 ^d^	n.d.	1.3434 ± 0.0004 ^b^	0.4790 ± 0.0007 ^d^	0.460 ± 0.005 ^d^	0.951 ± 0.005 ^c^
Total phenolic compounds	3.93 ± 0.05 ^b^	n.d.	4.1 ± 0.1 ^a^	1.61 ± 0.03 ^d,e^	n.d.	1.689 ± 0.008 ^d^	1.818 ± 0.007 ^c^	0.536 ± 0.005 ^f^	1.53 ± 0.01 ^e^

n.d.—not detected. tr.—traces. Calibration curves used for quantification: (-)-catequin (*y* = 84,950*x* − 23,200, *R*^2^ = 0.999, LOD = 0.17 μg/mL; LOQ = 0.68 μg/mL, peak 1); *p*-hydroxybenzoic acid (*y* = 208,604*x* + 173056, *R*^2^ = 0.9995, LOD = 1.37 μg/mL; LOQ = 4.15 μg/mL, peak 3); and quercetin 3-*O*-glucoside (*y* = 34,843*x* − 160,173; *R*^2^ = 0.9998; LOD = 0.21 μg/mL; LOQ = 0.71 μg/mL, peaks 2, 4, 5, 6, 7 and 8). ANOVA analysis—In each row different letters mean significant differences (*p* < 0.05).

**Table 4:** Antioxidant activity of the byproducts of three pumpkin genotypes from Portugal, obtained through cell-based assays.

Pumpkin Genotype from Portugal	Part	OxHLIA 60 min IC_50_ ^1^, μg/mL	TBARS IC_50_ ^1^, μg/mL
Butternut Squash	Peel	88 ± 3 ^c^	7461 ± 315 ^b^
	Seeds	59 ± 6 ^d^	185 ± 7 ^h^
	Fibrous strands	44 ± 4 ^d^	6887 ± 53 ^c^
Common Pumpkin	Peel	90 ± 3 ^c^	3921 ± 33 ^e^
	Seeds	43 ± 3 ^d^	756 ± 27 ^g^
	Fibrous strands	365 ± 13 ^a^	6375 ± 68 ^d^
Kabocha Squash	Peel	209 ± 10 ^b^	7765 ± 31 ^a^
	Seeds	46 ± 2 ^d^	164 ± 8 ^h^
	Fibrous strands	96 ± 2 ^c^	1568 ± 53 ^f^
Trolox		21.8 ± 0.2 ^e^	139 ± 5 ^h^

^1^ IC_50_: Extract concentration that inhibits lipid peroxidation by 50%. ANOVA analysis—In each column, different letters mean significant differences (*p* < 0.05).

**Table 5:** Antioxidant activity of the byproducts of three pumpkin genotypes from Algeria, obtained through cell-based assays.

Pumpkin Genotype from Algeria	Part	OxHLIA 60 min IC_50_ ^1^, μg/mL	TBARS IC_50_ ^1^, μg/mL
Butternut Squash	Peel	588 ± 18 ^a^	4569 ± 277 ^a^
	Seeds	115 ± 6 ^f^	573 ± 31 ^e^
	Fibrous strands	257 ± 13 ^d^	3508 ± 91 ^b^
Gold Nugget Pumpkin	Peel	362 ± 8 ^b,c^	3123 ± 136 ^c^
	Seeds	n.d. ^2^	91 ± 4 ^f^
	Fibrous strands	566 ± 13 ^a^	3659 ± 199 ^b^
Musquée de Provence	Peel	335 ± 4 ^c^	2123 ± 101 ^d^
	Seeds	400 ± 34 ^b^	549 ± 27 ^e^
	Fibrous strands	188 ± 2 ^e^	4385 ± 242 ^a^
Trolox		21.8 ± 0.2 ^g^	139 ± 5 ^f^

^1^ IC_50_: Extract concentration that inhibits oxidative hemolysis by 50%. ^2^ Not detected. ANOVA analysis: In each column, different letters mean significant differences (*p* < 0.05).

**Table 6:** Antibacterial activity of the byproducts of three pumpkin genotypes from Portugal.

	Butternut Squash						Common Pumpkin						Kabocha Squash						Streptomicin 1 mg/mL		Methicilin 1 mg/mL		Ampicillin 10 mg/mL	
	Seeds		Peel		Fibrous Strands		Seeds		Peel		Fibrous Strands		Seeds		Peel		Fibrous Strands							
	MIC	MBC	MIC	MBC	MIC	MBC	MIC	MBC	MIC	MBC	MIC	MBC	MIC	MBC	MIC	MBC	MIC	MBC	MIC	MBC	MIC	MBC	MIC	MBC
Gram-negative bacteria																								
*Enterobacter cloacae*	10	>10	10	>10	2.5	>10	10	>10	10	>10	>10	>10	10	>10	10	>10	10	>10	0.007	0.007	n.t.	n.t	0.15	0.15
*Escherichia coli*	>10	>10	10	>10	10	>10	10	>10	10	>10	>10	>10	10	>10	10	>10	10	>10	0.01	0.01	n.t.	n.t.	0.15	0.15
*Pseudomonas aeruginosa*	>10	>10	>10	>10	10	>10	>10	>10	>10	>10	>10	>10	10	>10	10	>10	10	>10	0.06	0.06	n.t.	n.t.	0.63	0.63
*Salmonella enterica*	>10	>10	10	>10	10	>10	10	>10	10	>10	>10	>10	10	>10	10	>10	10	>10	0.007	0.007	n.t.	n.t.	0.15	0.15
*Yersinia enterocolitica*	10	>10	5	>10	5	>10	10	>10	10	>10	10	>10	10	>10	10	>10	5	>10	0.007	0.007	n.t.	n.t.	0.15	0.15
Gram-positive bacteria																								
*Bacillus cereus*	>10	>10	>10	>10	5	>10	10	>10	2.5	>10	10	>10	>10	>10	>10	>10	>10	>10	0.007	0.007	n.t.	n.t.	n.t.	n.t.
*Listeria monocytogenes*	>10	>10	>10	>10	5	>10	10	>10	10	>10	10	>10	>10	>10	10	>10	10	>10	0.007	0.007	n.t.	n.t.	0.15	0.15
*Staphylococcus aureus*	10	>10	>10	>10	10	>10	10	>10	5	>10	10	>10	10	>10	10	>10	10	>10	0.007	0.007	0.007	0.007	0.15	0.15

MIC: Minimum inhibitory concentration (mg/mL); MBC: Minimal bactericidal concentration (mg/mL). n.t: not tested.

**Table 7:** Antifungal activity of the byproducts of three pumpkin genotypes from Portugal.

	Butternut Squash						Common Pumpkin						Kabocha Squash						Ketoconazole	
	Seeds		Peel		Fibrous Strands		Seeds		Peel		Fibrous Strands		Seeds		Peel		Fibrous Strands			
	MIC	MFC	MIC	MFC	MIC	MFC	MIC	MFC	MIC	MFC	MIC	MFC	MIC	MFC	MIC	MFC	MIC	MFC	MIC	MFC
*Aspergillus brasiliensis*	10	>10	10	>10	5	>10	5	>10	10	>10	10	>10	10	>10	10	>10	10	>10	0.06	0.125
*Aspergillus fumigatus*	>10	>10	>10	>10	>10	>10	>10	>10	>10	>10	>10	>10	>10	>10	>10	>10	>10	>10	0.5	1

MIC: Minimum inhibitory concentration (mg/mL); MFC: Minimal fungicidal concentration (mg/mL).

**Table 8:** Antibacterial activity of the byproducts of three pumpkin genotypes from Algeria.

	Gold Nugget Pumpkin						Butternut Squash						Musquée de Provence						Streptomicin 1 mg/mL		Methicilin 1 mg/mL		Ampicillin 10 mg/mL	
	Seeds		Peel		Fibrous Strands		Seeds		Peel		Fibrous Strands		Seeds		Peel		Fibrous Strands							
	MIC	MBC	MIC	MBC	MIC	MBC	MIC	MBC	MIC	MBC	MIC	MBC	MIC	MBC	MIC	MBC	MIC	MBC	MIC	MBC	MIC	MBC	MIC	MBC
Gram-negative bacteria																								
*Enterobacter Cloacae*	5	>10	5	>10	>10	>10	5	>10	10	>10	10	>10	10	>10	>10	>10	>10	>10	0.007	0.007	n.t.	n.t	0.15	0.15
*Escherichia coli*	10	>10	10	>10	10	>10	10	>10	10	>10	10	>10	>10	>10	10	>10	>10	>10	0.01	0.01	n.t.	n.t.	0.15	0.15
*Pseudomonas aeruginosa*	>10	>10	>10	>10	>10	>10	>10	>10	>10	>10	>10	>10	>10	>10	>10	>10	>10	>10	0.06	0.06	n.t.	n.t.	0.63	0.63
*Salmonella enterica*	10	>10	10	>10	>10	>10	10	>10	10	>10	>10	>10	>10	>10	>10	>10	>10	>10	0.007	0.007	n.t.	n.t.	0.15	0.15
*Yersinia enterocolitica*	5	>10	5	>10	10	>10	5	>10	10	>10	>10	>10	10	>10	>10	>10	>10	>10	0.007	0.007	n.t.	n.t.	0.15	0.15
Gram-positive bacteria																								
*Bacillus cereus*	5	>10	>10	>10	>10	>10	2.5	>10	>10	>10	>10	>10	>10	>10	>10	>10	>10	>10	0.007	0.007	n.t.	n.t.	n.t.	n.t.
*Listeria monocytogenes*	2.5	>10	5	>10	10	>10	10	>10	>10	>10	5	>10	>10	>10	>10	>10	5	>10	0.007	0.007	n.t.	n.t.	0.15	0.15
*Staphylococcus aureus*	2.5	>10	5	>10	10	>10	5	>10	5	>10	10	>10	10	>10	5	>10	10	>10	0.007	0.007	0.007	0.007	0.15	0.15

MIC: Minimum inhibitory concentration (mg/mL); MBC: Minimal bactericidal concentration (mg/mL). n.t: not tested.

**Table 9:** Antifungal activity of the byproducts of three pumpkin genotypes from Algeria.

	Gold Nugget Pumpkin						Butternut Squash						Musquée de Provence						Ketoconazole	
	Seeds		Peel		Fibrous Strands		Seeds		Peel		Fibrous Strands		Seeds		Peel		Fibrous Strands			
	MIC	MFC	MIC	MFC	MIC	MFC	MIC	MFC	MIC	MFC	MIC	MFC	MIC	MFC	MIC	MFC	MIC	MFC	MIC	MFC
*Aspergillus brasiliensis*	5	>10	5	>10	10	>10	5	>10	10	>10	10	>10	5	>10	10	>10	10	>10	0.06	0.125
*Aspergillus fumigatus*	>10	>10	>10	>10	10	>10	>10	>10	>10	>10	10	>10	>10	>10	>10	>10	10	>10	0.5	1

MIC: Minimum inhibitory concentration (mg/mL); MFC: Minimal fungicidal concentration (mg/mL).

## Data Availability

Not applicable.
